# Associations between domestic violence and poor pregnancy outcomes in taiwanese women: a nested case–control study

**DOI:** 10.1186/s12905-023-02602-x

**Published:** 2023-09-01

**Authors:** Chia-Ling Lin, Jui-Chiung Sun, Chun-Ping Lin, Chi-Hsiang Chung, Wu-Chien Chien

**Affiliations:** 1 Department of Nursing, Chang Gung University of Science and Technology, Taoyuan, Taiwan; 2 Department of Nursing, Shu-Zen Junior College of Medicine and Management, Kaohsiung, Taiwan; 3 School of Public Health, National Defense Medical Center, Taipei, Taiwan; 4 Department of Medical Research, Tri-Service General Hospital, No.325, Section 2, Cheng-Kung Road, Neihu District, Taipei City, 114 Taiwan

**Keywords:** Abortion, Domestic violence, Nested case–control study, Poor pregnancy outcomes, Premature delivery, Stillbirth

## Abstract

**Background and aims:**

Domestic violence (DV) are one of the important risk factors for women’s health outcomes. The aim of this study was explored the risk of DV association with the poor pregnancy outcomes (PPOs), including premature delivery, abortion, and stillbirth.

**Methods:**

A nested case–control study was applied. Data from the Taiwan National Health Insurance Research Database were collected from 2000 to 2015. A total of 41,730 participants were included in this study, including 8,346 participants in the case group and 33,384 age- and index year-matched control group. Assessments of DA and PPOs were determined according to the International Classification of Diseases, 9th Revision. We conducted a conditional logistic regression analysis to estimate the effect of DV on PPOs.

**Results:**

The mean age was 35.53 in the 41,730 female participants. The overall incidence rate of PPOs of the participants, who had experienced DV, was 84.05 per 100,000 person-years. which was significantly higher than that for the controls (18.19 per 100,000 person-years). The risk of PPOs was higher in the participants who had experienced DV than in the controls (adjusted odds ratio [AOR] = 3.31; 95% confidence interval [CI] [95% CI]: 2.83–3.86), including for premature delivery (AOR = 3.57; 95% CI: 3.05–4.17), abortion (AOR = 3.31; 95% CI: 2.83–3.86) and stillbirth (AOR = 2.98; 95% CI: 2.55–3.47). The results showed that the longer a participant has been suffering DV, the risk of PPOs was higher.

**Conclusions:**

Present results reaved the risk of PPOs associated with DV. Especially, the longer a woman has been experiencing DV, the risk of PPOs was higher, showed a dose–response effect.

**Supplementary Information:**

The online version contains supplementary material available at 10.1186/s12905-023-02602-x.

## Introduction

Domestic violence (DV) is a significant risk factor that negatively impacts women’s lives [1, 2]. Recent studies indicate that approximately 4.68–25% of women have reported experiencing some form of DV [3, 4]. More specifically, research has found that the prevalence of DV during pregnancy ranges from 1.2 to 7.3% in certain countries [5–7]. DV is a significant public health issue that detrimentally affects fetal and pregnancy outcomes [5, 6, 8].

Previous studies have examined the association between DV during pregnancy and adverse birth outcomes, including abortion, stillbirth, low birth weight, premature birth, and neonatal death [5, 8, 9]. While most research has shown a positive association, some studies have not found such a link [10–12].

Most published findings indicate that DV is a risk factor for adverse fetal and pregnancy outcomes in fertile women. However, few studies have examined the association between abnormal pregnancy outcomes and the intensity and frequency of DV. Therefore, the aim of this study was to investigate the association between DV and poor pregnancy outcomes (PPOs), including premature delivery, abortion, and stillbirth.

## Method

### Data source

We used Taiwan National Health Insurance Research Database (NHIRD) managed by government, which involved medical treatment information for the public in Taiwan; the NHIRD is population-based claims database covering over 99.9% of Taiwan’s population [13]. In this study, disease diagnoses were collected from different datasets, which are categorized based on the International Classification of Diseases, Ninth Revision, Clinical Modification (ICD-9-CM). The NHIRD offers comprehensive information on the medical utilization of almost all pregnant women and birth register records in Taiwan, providing a highly reliable opportunity to investigate the relationship between DV and PPOs [14].

### Study Design and sampled participants

This study employed a population-based nested case-control design. The case group was defined as participants who experienced PPOs identified by ICD-9-CM codes for premature delivery (ICD-9-CM code 644.21), abortion (ICD-9-CM code 634.9), or stillbirth (ICD-9-CM code 656.43) and at least once with principal or secondary diagnoses indicating PPOs between 2000 and 2015.

**Fig. 1:**
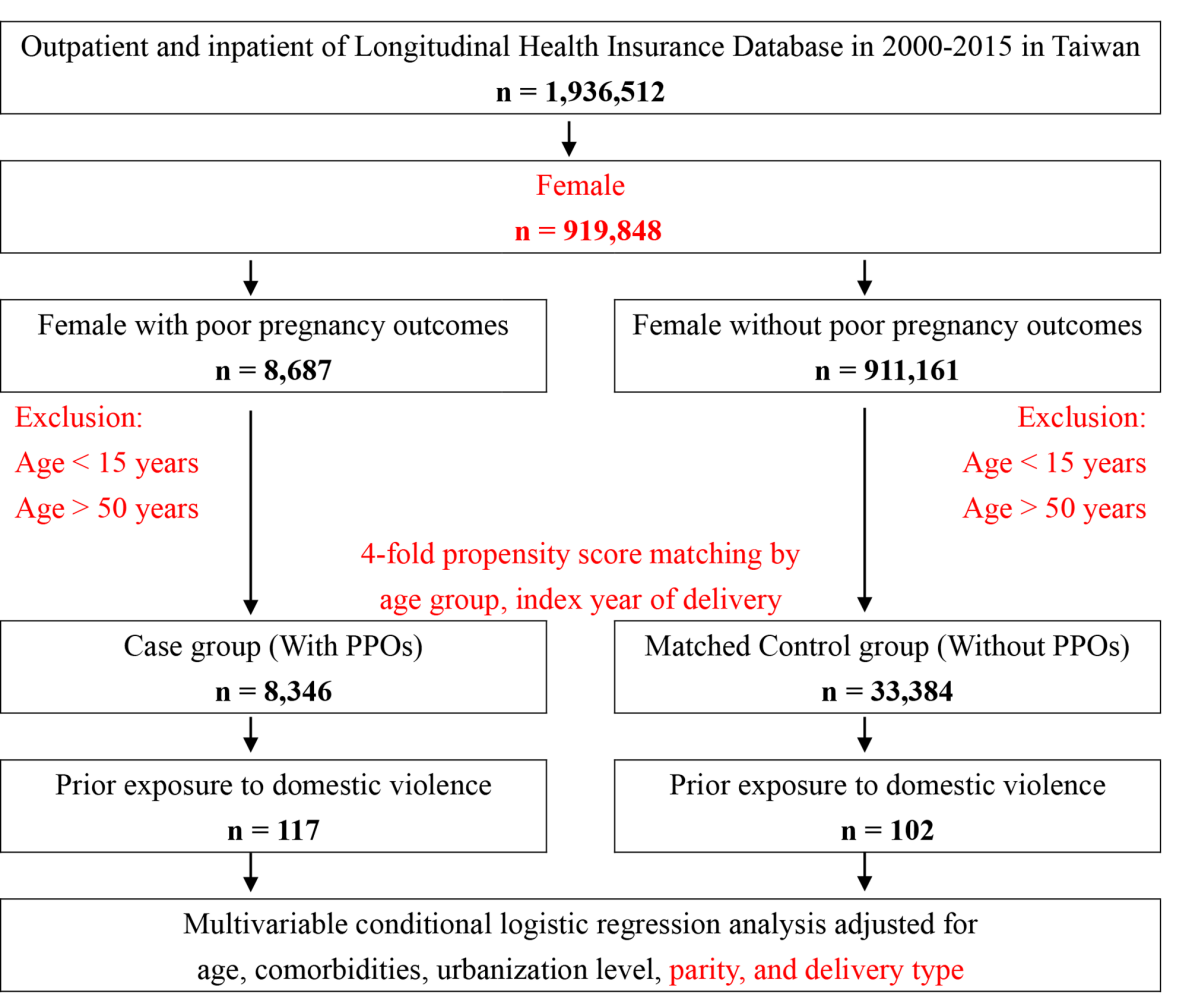
The flowchart of nested case-control study from National Health Insurance Research Database in Taiwan

The control group was selection of the participants with a control-to-case ratio of 4:1 used propensity scores (Fig. 1), and without PPOs and frequency-matched by age group and index year of delivery. The exclusion criteria were women aged < 15 and > 50 years.

### Definition of DV

This study selected participants who had experienced DV based on ICD-9-CM codes (995. xx; E967.x) between 2000 and 2015.

### Definition of comorbidity

Comorbidities were defined as at least one of the following primary or secondary diagnoses: type 1 diabetes, type 2 diabetes, anxiety, cardiovascular disease, depression, eclampsia, gestational diabetes mellitus, obesity, preeclampsia, syphilis, thalassemia, and alcoholism according to ICD-9-CM codes. Additionally, based on previous study, urbanization levels 1 (highest) to 4 (lowest) were defined [15].

### Data analysis

The SPSS (version 22, IBM Corp., Armonk, New York) was applied in this study to execute all data analyses. The rate of the outcomes was calculated by the total follow-up duration (person-years). The χ^2^ test was applied to estimate differences in the categorical variables, and Student’s t test was used to calculate the differences in the continuous variables. Conditional logistic regression was performed to estimate the correlation between previous DV history and the occurrence of PPOs among female participants. Data are presented as odds ratios (ORs) with 95% confidence intervals (CIs). Adjusted odds ratio (AOR) included age, comorbidities, urbanization level, parity, and delivery type. The P values provided are two-sided, with the level of significance at 0.05.

## Results

### Demographic data

As shown in Table 1, there were 41,730 participants in this study (8,346 cases and 33,384 controls), with a mean age of 35.53±19.17 years. Overall, the mean of DV frequency was 3.26±4.28 times. Participants in the PPO group got a higher prevalence of T2DM, GDM, preeclampsia, eclampsia, CVD, anxiety, depression, syphilis, and alcoholism. In the case group, those accounted for most of the participants were parity 1 and had a caesarean section delivery.

**Table 1:** Characteristics of case group and matched control group

PPOS	Overall (n = 41,730 )		With, *Case group* (n = 8,346 )		Without, *Control group* (n = 33,384 )		*P*
Variables	n	%	n	%	n	%	
**DV**	219	0.52	117	1.40	102	0.31	< 0.001
**DV frequency (mean ± SD)**	3.26 ± 4.28		3.86 ± 4.37		3.11 ± 4.25		< 0.001
**Age (y) (mean ± SD)**	35.53 ± 19.17		35.72 ± 19.20		35.48 ± 19.16		0.306
**Age group (y)**							0.999
15–19	1,670	4.00	334	4.00	1,336	4.00	
20–24	4,860	11.65	972	11.65	3,888	11.65	
25–29	4,440	10.64	888	10.64	3,552	10.64	
30–34	7,135	17.10	1,427	17.10	5,708	17.10	
35–39	7,865	18.85	1,573	18.85	6,292	18.85	
40–44	7,800	18.69	1,560	18.69	6,240	18.69	
45–49	7,960	19.08	1,592	19.08	6,368	19.08	
**Comorbidities**							
T1DM	825	1.98	151	1.81	674	2.02	0.235
T2DM	7,329	17.56	1,577	18.90	5,752	17.23	< 0.001
GDM	670	1.61	281	3.37	389	1.17	< 0.001
Pre-eclampsia	568	1.36	491	5.88	77	0.23	< 0.001
Eclampsia	395	0.95	351	4.21	44	0.13	< 0.001
CVD	3,922	9.40	1,026	12.29	2,896	8.67	< 0.001
Thalassemia	211	0.51	44	0.53	167	0.50	0.731
Obesity	321	0.77	77	0.92	244	0.73	0.080
Anxiety	2,570	6.16	1,556	18.64	1,014	3.04	< 0.001
Depression	2,108	5.05	1,322	15.84	786	2.35	< 0.001
Syphilis	102	0.24	39	0.47	63	0.19	< 0.001
Alcoholism	99	0.24	29	0.35	70	0.21	0.031
**Urbanization level**							0.293
1 (The highest)	10,460	25.07	2,125	25.46	8,335	24.97	
2	11,144	26.71	2,264	27.13	8,880	26.60	
3	9,962	23.87	1,986	23.80	7,976	23.89	
4 (The lowest)	10,164	24.36	1,971	23.62	8,193	24.54	
**Parity**							< 0.001
1	25,294	60.61	5,562	66.64	19,732	59.11	
≧ 2	16,436	39.39	2,784	33.36	13,652	40.89	
**Delivery type**							< 0.001
Normal spontaneous delivery	21,523	51.58	4,003	47.96	17,520	52.48	
Cesarean section	20,207	48.42	4,343	52.04	15,864	47.52	

PPOs: poor pregnancy outcomes; DV: domestic violence; T1DM: Type 1 diabetes mellitus; T2DM: Type 2 diabetes mellitus; GDM: gestational diabetes mellitus; CVD: cardiovascular disease

### Factors of PPOs stratified by using conditional logistic regression

Table 2 presents a significantly higher risk of PPOs in the participants with DV experience than in the controls (AOR = 3.31; 95% CI: 2.83–3.86) after adjustment for age group, comorbidities, urbanization level, parity, and delivery type. When the age group was divided, there was a significantly higher risk of PPOs in participants aged 45–49 compared with in the other age groups (AOR = 3.70, 95% CI: 3.17–4.32).

**Table 2:** Factors of poor pregnancy outcomes stratified by variables listed in the table by using conditional logistic regression

Stratified	With PPOs			Without PPOs			With PPOs vs. Without PPOs *(Control)*		
	DV exposure	PYs	Rate	DV exposure	PYs	Rate	AOR	95% CI	*P*
**Overall**	117	139,203.50	84.05	102	560,796.41	18.19	3.31	2.83–3.86	< 0.001
Age group (y)*									
15–19	9	10,367.33	86.81	9	41,361.82	21.76	2.84	2.44–3.32	< 0.001
20–24	11	15,172.09	72.50	11	63,004.34	17.46	3.02	2.59–3.52	< 0.001
25–29	16	18,145.00	88.18	15	74,762.44	20.06	3.17	2.71–3.70	< 0.001
30–34	20	23,478.70	85.18	18	94,448.32	19.06	3.20	2.74–3.73	< 0.001
35–39	18	20,359.71	88.41	18	92,804.60	19.40	3.54	3.03–4.13	< 0.001
40–44	19	22,881.18	83.04	16	94,377.51	16.95	3.55	3.05–4.15	< 0.001
45–49	24	28,799.49	83.33	15	100,037.39	14.99	3.70	3.17–4.32	< 0.001
**Comorbidities**									
Without	24	52,229.87	45.95	20	205,436.02	9.74	2.69	2.30–3.14	< 0.001
1–2	36	37,066.05	97.12	36	159,851.62	22.52	3.22	2.76–3.75	< 0.001
≧3	57	49,907.59	114.21	46	195,508.77	23.53	3.72	3.19–4.34	< 0.001
**Urbanization level**									
1	31	35,329.08	87.75	24	142,140.13	16.88	3.75	3.21–4.38	< 0.001
2	35	41,588.61	84.16	27	162,036.15	16.66	3.59	3.08–4.19	< 0.001
3	22	30,141.23	72.99	20	119,421.24	16.75	3.08	2.64–3.60	< 0.001
4	29	32,144.58	90.22	31	137,198.89	22.59	2.88	2.47–3.37	< 0.001
**Parity**									
1	59	48,474.29	121.71	52	300,105.67	17.33	3.24	2.78–3.78	< 0.001
≧ 2	58	90,729.21	63.93	50	260,690.74	19.18	3.37	2.89–3.94	< 0.001
**Delivery type**									
Normal spontaneous delivery	42	57,050.02	73.62	49	269,510.68	18.18	3.018	2.59–3.52	< 0.001
Cesarean section	75	82,153.48	91.29	53	291,285.73	18.20	3.533	3.03–4.12	< 0.001

PPOs: poor pregnancy outcomes; DV: domestic violence; PYs: person years; Rate: per 100,000 PYs; AOR = adjusted Odds Ratio; CI = confidence interval

Interaction term (Age group × Comorbidities): *P* = 0.353;Interaction term (Age group × Parity): *P* = 0.261

AOR indicates adjustment for age group, comorbidities, urbanization level, parity, and delivery type

*We conducted statistical testing between the groups and found that there was a significantly higher risk of PPOs in subjects aged 45–49 compared than in other age groups

### Factors of PPO subtypes by using conditional logistic regression

Table 3 indicates that the risk of PPO subtypes-premature delivery, abortion, and stillbirth-was higher (AOR = 3.57; 95% CI: 3.05–4.17; AOR = 3.31; 95% CI: 2.83–3.86; AOR = 2.98; 95% CI: 2.55–3.47, respectively) in the participants who had experienced DV than in the controls according to the conditional logistic regression analyses.

**Table 3:** Factors of poor pregnancy outcomes subtypes by using conditional logistic regression

PPOs	With PPOs			Without PPOs			With PPOs vs. Without PPOs *(Control)*		
	DV exposure	PYs	Rate	DV exposure	PYs	Rate	AOR	95% CI	*P*
Premature delivery	49	139,561.56	35.11	41	561,125.37	7.31	3.57	3.05–4.17	< 0.001
Abortion	38	139,629.61	27.21	32	561,167.89	5.70	3.31	2.83–3.86	< 0.001
Stillbirth	30	139,665.86	21.48	29	561,183.17	5.17	2.98	2.55–3.47	< 0.001

AOR = adjusted Odds Ratio; CI = confidence interval; PPOs: poor pregnancy outcomes; DV: domestic violence; PYs: person years; Rate: per 100,000 PYs;

AOR indicates adjustment for age group, comorbidities, urbanization level, parity, and delivery type

### Factors of PPO among different DV exposure period and frequency by using conditional logistic regression

Table 4 presents the duration of DV from the first exposure to the last exposure before a PPO diagnosis; women with a longer duration of DV had a higher risk of PPOs. Table 5 demonstrates that higher frequency of DV is associated with an increased risk of PPOs.

**Table 4:** Factors of poor pregnancy outcomes among different violence exposure period by using conditional logistic regression

Exposure DV duration*	With PPOs vs. Without PPOs *(Control)*		
	AOR	95% CI	*P*
< 1 year	2.87	2.69–3.62	< 0.001
≧1 year, < 2 years	3.32	2.91–3.90	< 0.001
≧2 years	3.42	3.00–4.03	< 0.001

AOR: adjusted Odds Ratio; CI: confidence interval; DV: domestic violence

^*^ Exposure DV duration indicated the first violence exposure to the last one before poor pregnancy outcomes diagnosis

AOR indicates adjustment for age group, comorbidities, urbanization level, parity, and delivery type

**Table 5:** Factors of poor pregnancy outcomes among different violence exposure frequency by using conditional logistic regression

DV frequency	With PPOs vs. Without PPOs *(Control)*		
	AOR	95% CI	*P*
1	2.04	1.35–3.37	< 0.001
2	3.39	2.98–4.01	< 0.001
≧3	4.01	3.42–5.86	< 0.001

AOR: adjusted Odds Ratio; CI: confidence interval; DV: domestic violence

AOR indicates adjustment for age group, comorbidities, urbanization level, parity, and delivery type

## Discussion

Our results demonstrated an association between DV and PPOs. Specifically, women who experienced DV had a 3.31 times higher risk of PPOs compared to controls. These findings are consistent with previous studies [8, 16]. Furthermore, there is a positive correlation between the duration and frequency of DV exposure in women and their risk of experiencing PPOs. A previous study indicated a dose-response relationship between the frequency of DV during pregnancy and the risk of premature birth [17]. Based on our comprehensive literature review, this study is the first to investigate the association between the intensity of DV and PPOs using a nested case-control design and longitudinal observation.

DV involves multiple complex factors and causes adverse health impacts in the female population. The true pathway by which DV increases the risk of PPOs remains unknown, prior reports have provided some reasons those may describe these results. First, previous animal research demonstrated that the repeated exposure of pregnancy rats to stress may change hypothalamic–pituitary–adrenal (HPA) function [18], and alter the corticotrophin-releasing hormone (CRH) concentration, could cause labour and limit uteroplacental perfusion [19]. Second, DV not only affects the reproductive health of women but also has fatal and nonfatal negative impact for the developing foetus due to the direct physical trauma from the abuse [16]. Third, women who experienced DV had a 25% decrease in the use of adequate antenatal care and a 20% decrease in the use of skilled delivery care, suggesting that when DV is underrecognized, women who experienced physical violence that results in injury may avoid medical use due to feelings of shame, feelings of dishonour, or fear of repercussions, thus decreasing their use of adequate antenatal care services [20], above this evidence supports that DV increases adverse impacts on birth outcomes.

In this study, a stratified method was employed to control for the effect of age, revealing that the highest prevalence of PPOs was observed in the age group of 45–49 years old. This can be attributed to the fact that the risk of adverse pregnancy outcomes tends to increase with age [21]. Furthermore, this study established a positive correlation between prolonged exposure to DV and the risk of PPOs. It should be noted that older women of PPOs may potentially have experienced a longer duration of DV exposure.

Generally, DV can have both direct and indirect impacts on pregnancy outcomes. Some similarities included DV can individual a pregnant woman to emotional stress, potentially affecting the developing fetus and pregnancy outcomes. Secondly, DV can endanger the health of pregnant women, and physical abuse or trauma associated with DV can pose risks to their well-being, consequently impacting the fetus outcomes [9, 22]). However, there may still be some differences in the impact of DV on fetal outcomes at different gestational weeks. For example, DV may influence labor outcomes through stress responses, such as the release of vasoconstrictors, cortisol, and prostaglandins, potentially resulting in premature uterine contractions [9, 23]. Additionally, pregnancy can be terminated through induced abortion in cases involving DV, depression, post-traumatic stress disorder, psychological distress, or suicidal tendencies [24]. Thirdly, DV can lead to reduced nutritional intake, inadequate antenatal care, and an increased risk of stillbirth [22, 25].

Our results highlight the key point of health organizations in DV screening and support for women with a history of DV who occur to experience a disproportionate burden of high-risk pregnancy events. Other studies have made similar viewpoints [8]. Health care providers are often the first line of help for people experiencing DV. Without keen observation, professional training and relevant experience, the experience of the person is ignored. Thus, we suggest that health care providers provide a safe and private space for diagnosis, treatment, and interviews, and connect with security guards, focusing increasing the person’s sense of security.

The effective identification of the risk and protective factors for DV is critical to addressing the problem early. This will help reduce the impact of DV and help develop prevention and control strategies. Health care providers play a key role as a resource linker. Through assessment, early detection, and linkage of related resources, women’s sense of security can be enhanced, and their physical and mental anxiety and uncertainty can be reduced, becoming one of women’s support systems, to prevent the recurrence of DV and its irreversible health risks to pregnant women [26]. Some evidence suggests that home visits and behavioural counselling interventions that address multiple risk factors may lead to reduced DV among pregnant women and recommends that clinicians screen for DV and provide ongoing support services [27, 28].

### Strengths and limitations

This study revealed that maternal exposure to DV was associated with a significantly increased risk of PPOs. These findings emphasize the relationship between the frequency and intensity of DV and the increased risk of PPOs. This research is one of the few to apply diagnostic criteria for DV and PPOs in the analysis of a population-based nested case–control study. However, several limitations of this study should be addressed. First, the NHIRD does not provide detailed information on socioeconomic factors, such as the level of education, level of income, or use of alcohol and drugs. Second, the NHIRD data does not included detailed information on the type of DV. Third, the study participants were selected using medical records from the NHIRD. Data for participants who had experienced DV but did not undergo treatment or assessment in the medical institution were not recorded in the NHIRD, the prevalence of exposure to DV during pregnancy in our study (0.52%) was lower than that found in previous study [5]; therefore, there may be selection bias in underestimating the prevalence of DV in the results. Additionally, the severity of DV could not be recorded in the databank. Some participants might have disclosed their experiences to an interviewer due to stigma, ongoing trauma, or safety concerns in the hospital and data could not be recorded in the NHIRD; not all potential confounders were accounted for in this analysis.

## Conclusions

These findings are important from the maternal health policy and human rights perspectives. Women’s exposure to DV is associated with adverse consequences for birth outcomes. A decrease in the burden of DV against women is not only likely to improve the health and quality of life but is also likely to improve poor delivery events.

Present study supported that DV is significant factor for poor birth outcomes in women. We recommend early detection via nursing assessment for the signs of family violence and providing the properly protect care for women.

### Electronic supplementary material

Below is the link to the electronic supplementary material.

Supplementary Material 1

## Data Availability

All data generated or analysed during this study are included in this published article.

## Data Availability

Data are from the National Health Institute Research Database, which is available to researchers in Taiwan and has been extensively used in epidemiologic studies. Use is allowed for academic purpose only after proof. Thus, the data cannot be made publicly available.
