# Supplementary material for: Associations between domestic violence and poor pregnancy outcomes in taiwanese women: a nested case–control study
Source: BMC Womens Health. 2023 Sep 1;23:465. doi: 10.1186/s12905-023-02602-x (PMC10474664; doi:10.1186/s12905-023-02602-x)
Supplement: Supplementary file 1 — Supplementary Material 1 [file 12905_2023_2602_MOESM1_ESM.doc]

Supplementary File

Supplementary Table 1. The last DV exposure before the first PPOs by using conditional logistic regression

|  | **With PPOs *vs.* Without PPOs** *(control)* | | |
| --- | --- | --- | --- |
| **The last DV exposure before the first PPOs** | **AOR** | **95% CI** | ***P*** |
| < 1 year | 3.41 | 2.97 - 3.98 | <0.001 |
| ≧1 year, < 2 years | 3.32 | 2.84 - 3.86 | <0.001 |
| ≧2 years | 3.29 | 2.76 - 3.70 | <0.001 |

AOR: adjusted Odds Ratio; CI: confidence interval; DV: domestic violence

AOR indicates adjustment for age group, comorbidities, urbanization level, parity, and delivery type.
